# *Z. morio* Hemolymph Relieves *E. coli*-Induced Mastitis by Inhibiting Inflammatory Response and Repairing the Blood–Milk Barrier

**DOI:** 10.3390/ijms232113279

**Published:** 2022-10-31

**Authors:** Yunjing Zou, Xue Wang, Jiajia Xu, Shenghua Wang, Shuxian Li, Yaohong Zhu, Jiufeng Wang

**Affiliations:** College of Veterinary Medicine, China Agricultural University, Beijing 100193, China

**Keywords:** mastitis, inflammasome, blood–milk barrier, *Escherichia coli*, *Z. morio* hemolymph

## Abstract

*Escherichia coli* (*E. coli*) is a major environmental pathogen causing coliform mastitis, characterized by cell death and mammary tissue damage. Our previous study has shown the antimicrobial effect of *Zophobas morio* (*Z. morio*) hemolymph against mastitis pathogens. In this study, we established *E. coli*-induced cellular and animal models for mastitis, aiming to evaluate the protective effect of *Z. morio* hemolymph against *E. coli*-induced mastitis in vivo and in vitro. In mice with *E. coli*, *Z. morio* hemolymph attenuated bacterial burden and histopathological impairment, reduced the production of interleukin (IL)-1β, IL-18, tumor necrosis factor-α (TNF-α) and the ratio of CD4^+^ T/CD8^+^ T, and increased the production of IL-2 triggered by *E. coli*. *Z. morio* hemolymph also enhanced the integrity of the blood-milk barrier in *E. coli*-induced mastitis. In *E. coli*-stimulated porcine mammary epithelial cells, *Z. morio* hemolymph inhibited *E. coli*-induced inflammatory responses and upregulated tight junction proteins (ZO-1, Claudin-3 and Occludin). Moreover, we found that the anti-inflammatory effect of *Z. morio* hemolymph was mediated by inhibiting *E. coli*-induced NLRP3 inflammasome assembly, Caspase-1 activation, and reversing the inhibitory effect of *E. coli* on autophagy. Besides, *Z. morio* hemolymph augmented ATG5/ATG16L1-mediated autophagy activation, negatively regulated NLRP3 inflammasome activation. Our results reveal that *Z. morio* hemolymph alleviates *E. coli*-induced mastitis via lessening the inflammatory response by regulating the NLRP3 and ATG5/ATG16L1 signaling pathway, as well as repairing the blood-milk barrier.

## 1. Introduction

Coliform mastitis (CM) is one of the most important symptoms of postpartum dysagalactia syndrome (PDS), which is an economically relevant disease in postpartum sows that also severely affects the health, welfare, and performance of the piglets [1,2]. *Escherichia coli* (*E. coli*), one of the most prevalent mastitis-causing pathogens, is most commonly isolated from milk of PDS-affected sows [3,4], triggering swift and decisive inflammation in mammary glands and mammary epithelial cells [5,6]. Not surprisingly, antibiotic treatment is still the main strategy for treating acute mastitis, which seems to be effective against bacteria but increases the risk of transmission of antimicrobial resistance to commensal and opportunistic bacteria, posing threats to public health security and increasing veterinary care costs [7]. Thus, it is urgent to find and develop new antibiotic alternatives for the treatment of mastitis.

Insect antimicrobial peptides (AMPs) have gained special attention as an alternative to antibiotics. They possess a broad range of antibacterial, antifungal, and antiviral activities, which also have the feature of high performance, least toxicity, and difficult to form drug resistance [8,9]. Insect AMPs provide the first line of defense against a variety of pathogens [10,11,12]. Early studies reported that when lepidoptera larvae were attacked by low-dose bacteria, their hemolymph could secrete antibacterial molecules to deal with bacterial infection [13]. Moreover, a study indicated that up-regulation of antimicrobial peptides in bovine mammary tissue was effective in enhancing host innate immune response to mastitis, indicating that insect AMPs can be exploited as potential drugs in treating mastitis [14]. Interestingly, in our recent studies, *Z. morio* hemolymph showed the antimicrobial effect against a variety of mastitis-causing pathogens, including *E. coli* [15]. Therefore, we speculate that *Z. morio* hemolymph may have a protective effect on mastitis.

During a microbial infection, the host immune system gets activated. Increasing evidence has shown inflammasomes play a key role in driving host innate immune responses. The biochemical function of inflammasomes is to active Caspase-1 cleaves, which triggers the release of interleukin (IL)-1β and IL-18, as well as pyroptosis [16,17,18,19]. Generally, activation of inflammasome facilitates host defense against pathogenic infections. However, excessive inflammasome activation results in disorders of autoimmune and metabolic [20]. Research indicates that inhibition of NLRP3 inflammasome activation evidently ameliorates the severity of mastitis [21]. Furthermore, our previous study showed that attenuating the activation of NLRP3 inflammasome could improve *E. coli*-induced inflammatory damage in mammary epithelial cells [22]. Together, NLRP3 inflammasome can be a potential therapeutic target for mastitis. Meanwhile, several studies have analyzed that autophagy negatively regulates inflammasome activation. For example, autophagy activation inhibited the production of IL-1β and enhanced the degradation of inflammasome [23], the deletion of autophagic protein ATG16L1 in macrophages isolated from the mouse led to the activation of NLRP3 inflammasome and an obvious elevation of IL-1β and IL-18 [24]. However, the role of autophagy in regulating the immune response and inflammation to resist *E. coli*-induced porcine mastitis needs to be elucidated.

The blood–milk barrier is an important physical barrier for the mammary gland and is mainly composed of tight junctions (TJs) [25]. Recent findings indicated that lipopolysaccharide (LPS) could cause an early and acute mammary inflammation and lead to disruption of integrity of the blood-milk barrier, which is associated with the compositional changes of TJ proteins. Meanwhile, bacterial infections or inflammatory responses are exacerbated when the blood-milk barrier is weak or absent [26,27,28]. However, the role of the blood–milk barrier in *E. coli*-induced porcine mastitis is not known. The protective role of the blood–milk barrier during *Z. morio* hemolymph action needs to be explored.

Therefore, we hypothesize that *Z. morio* hemolymph ameliorates *E. coli*-induced repairs the blood–milk barrier and inflammatory response by inhibiting NLRP3 inflammasome activation and mediating autophagy activity. In the present study, the protective effects and the molecular mechanisms of *Z. morio* hemolymph were investigated by assessing alterations in the integrity of the blood–milk barrier and the inflammatory response to *E. coli*.

## 2. Results

### 2.1. Z. morio Hemolymph Alleviates Pathological Injury of Mammary Gland in E. coli-Induced Mastitis

**Figure 1:**
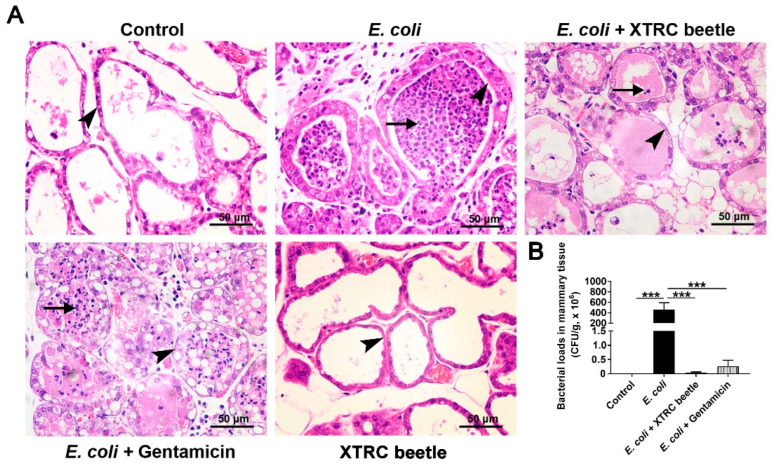
Effects of *Z. morio* hemolymph on mice mammary gland injury. (**A**) Histopathologic sections of mammary tissues. Short arrows and long arrows indicate neutrophils infiltration in acinar cavity and the thickening of mammary acinar walls, respectively. Scale bars, 50 μm. (**B**) Bacterial burden in the mammary tissues. XTRC beetle stands for *Z. morio* hemolymph-treatment. The data from each group are presented as the mean ± SEM (*n* = 8 per group). *** *p* < 0.001.

To screen the effective concentration of *Z. morio* hemolymph (Figure S1) and study the protective effect of *Z. morio* hemolymph against mastitis, the histological and morphological characteristics of mammary gland were assessed by H&E staining. There were no pathological injuries of mammary gland from the control group (Figure 1A). At 27 h post-infection, the mammary glands from *E. coli* group revealed severe histopathological changes, which mainly manifested as thickening of mammary gland alveolar walls and a massive recruitment of neutrophils infiltration. However, these pathological injuries were markedly alleviated by treatment with *Z. morio* hemolymph or gentamicin (Figure 1A). Consistently, the number of *E. coli* colonization in the mammary gland was 5.2 × 10^8^ ± 1.73 × 10^5^ CFU (means ± SEM), whereas *Z. morio* hemolymph or gentamicin treatment efficiently reduced the number of *E. coli* colonization in the mammary gland of mice, respectively, and no *E. coli* was detected in the mammary gland from the control group (Figure 1B).

### 2.2. Z. morio Hemolymph Affects Peripheral Blood Parameters in E. coli-Induced Mastitis

**Figure 2:**
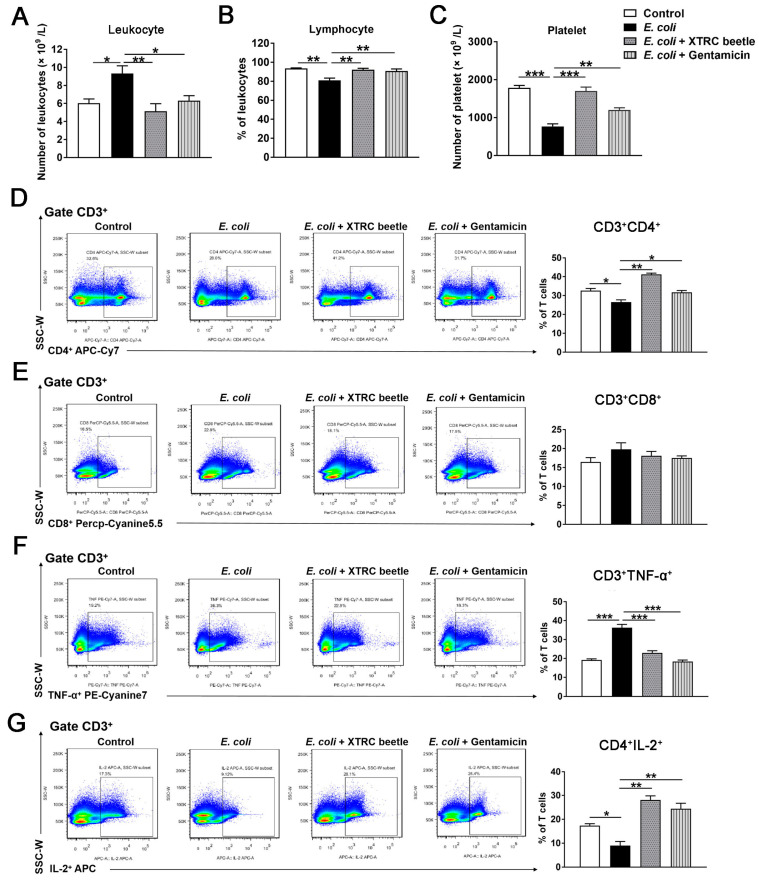
Effects of *Z. morio* hemolymph on peripheral blood parameters in *E. coli*-induced mastitis. (**A**) The total number of peripheral blood leukocytes. (**B**) The percentage of lymphocytes on blood leukocytes. (**C**) The total number of peripheral blood platelet. (**D**–**F**) The percentage of CD4^+^, CD8^+^ and TNF-a^+^ T cells among CD3^+^ T cells determined in peripheral blood lymphocytes of mice in different groups by flow cytometric analysis. The representative flow cytometry dot plot shows the gating strategy for CD4, CD8 and TNF-a expression in peripheral CD3^+^ T cells. (**G**) The percentage of IL-2+ T cells among CD3^+^CD4^+^ T cells determined in peripheral blood lymphocytes of mice in different groups by flow cytometric analysis. The representative flow cytometry dot plot shows the gating strategy for IL-2 expression in peripheral CD3^+^ T cells. XTRC beetle stands for *Z. morio* hemolymph-treatment. The data from each group are presented as the mean ± SEM (*n* = 8 per group). * *p* < 0.05, ** *p* < 0.01, *** *p* < 0.001.

Blood parameters can reflect the physiological state of the mice. To investigate the immunomodulatory effects of *Z. morio* hemolymph in *E. coli*-induced mice mastitis, the complete blood count (CBC) and T lymphocyte subsets expression in peripheral blood was analyzed. As shown, *E. coli* infection resulted in an increased count of peripheral blood leukocytes in the mammary gland, but this increase was attenuated by either *Z. morio* hemolymph or gentamicin treatment (Figure 2A). In contrast, the number of lymphocytes and platelet was decreased after *E. coli* infection compared with the control group, while *Z. morio* hemolymph or gentamicin treatment elevated the number of lymphocyte and platelet (Figure 2B,C). As expected, the result of flow cytometry showed that *E. coli* injection led to a remarkable reduction of CD4^+^ T cell counts compared with the control group, and *Z. morio* hemolymph or gentamicin administration significantly reversed this reduction in *E. coli*-induced mice mastitis (Figure 2D). Meanwhile, the CD8^+^ T cell counts in each group had no substantial changes regardless of *E. coli* injection, *Z. morio* hemolymph administration or Gentamicin treatment (Figure 2E). Furthermore, *E. coli* significantly increased the percentage of CD3^+^TNF-a^+^ T cells compared with the control group, whereas *Z. morio* hemolymph or gentamicin treatment led to a considerable decrease in the percentage of CD3^+^TNF-a^+^ T cells in comparison to *E. coli* group (Figure 2F). At 27 h post-infection, the percentage of CD3^+^CD4^+^IL-2^+^ T cells in mice of *E. coli* group was lower than that of the control, and a statistically dramatic increase of CD3^+^CD4^+^IL-2^+^ T cells percentage was observed in mice of *Z. morio* hemolymph or gentamicin treatment group relative to mice of *E. coli* group (Figure 2G).

### 2.3. Z. morio Hemolymph Inhibits the NLRP3 Signaling Pathway and Promotes ATG5/ATG16L1-Mediated Autophagy Signaling Pathway in E. coli-Induced Mastitis

**Figure 3:**
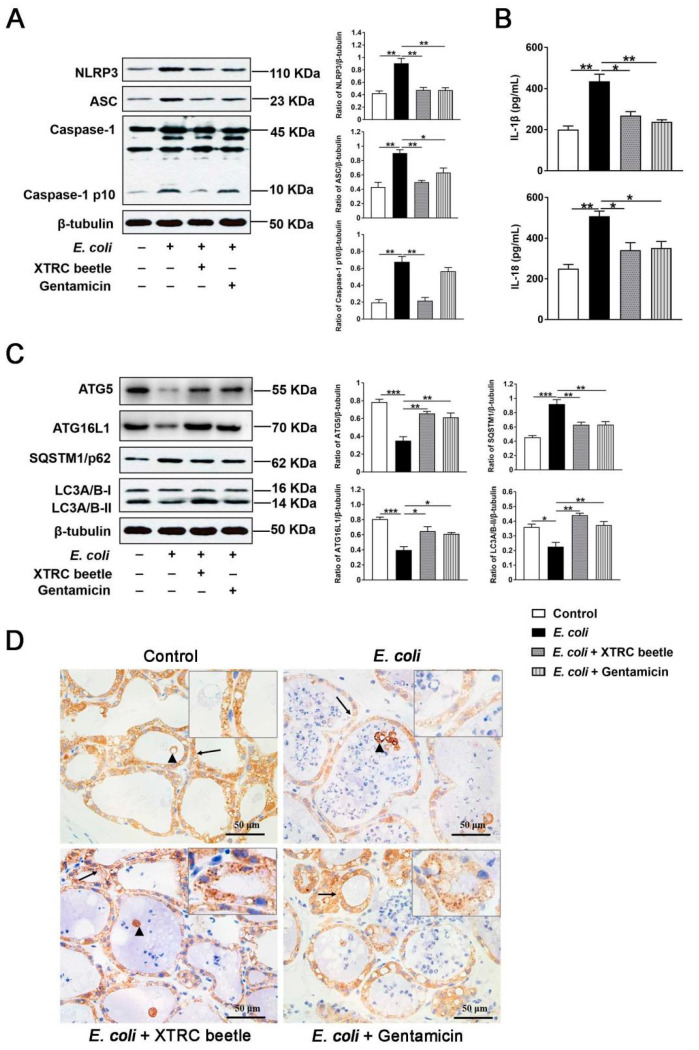
Effect of *Z. morio* hemolymph on the NLRP3 inflammasome and ATG5/ATG16L1-mediated autophagy signaling pathway in *E. coli*-induced mastitis. (**A**) Protein levels of NLRP3, ASC and Caspase-1 was measured using western blotting analysis. The right panel shows the protein quantification using ImageJ software (version 1.50). (**B**) Levels of IL-1β and IL-18 were measured by ELISA. (**C**) Protein levels of ATG5, ATG16L1, P62 and LC3A/B-II was measured using Western blotting analysis. The panel shows the protein quantification using ImageJ software (version 1.50). (**D**) Relative quantification and localization of LC3A/B-II (brown-yellow dots) expression by IHC. Black arrows indicate LC3A/B-II localized in the cytosol of MECs, black triangles indicate LC3A/B-II localized in the cytosol of MECs that exfoliated into the acinar lumen. XTRC beetle stands for *Z. morio* hemolymph-treatment. Data presented are means ± SEM (*n* = 8 per group). * *p* < 0.05, ** *p* < 0.01, *** *p* < 0.001.

NLRP3 and ATG5/ATG16L1-mediated autophagy signaling pathway plays an important role in regulating immune response to resist bacterial infections. To elucidate the anti-inflammatory mechanism of *Z. morio* hemolymph, the expression level of NLRP3 inflammasome and the secretion of IL-1β/IL-18 was determined by Western blotting and ELISA, respectively. As shown in Figure 3, *E. coli* injection significantly increased the protein levels of NLRP3, ASC and Caspase-1 p10, *Z. morio* hemolymph or gentamicin treatment markedly inhibited the protein levels of NLRP3, ASC and Caspase-1 p10 (Figure 3A). Furthermore, *E. coli* infection also resulted in a significant up-regulation in the protein levels of IL-1β and IL-18, while these effects were attenuated by *Z. morio* hemolymph or gentamicin administration (Figure 3B). In addition, *E. coli* injection reduced LC3A/B-II expression compared with the control group, while *Z. morio* hemolymph or gentamicin administration alleviated this reduction (Figure 3C). In contrast, *E. coli* injection increased P62 expression, while *Z. morio* hemolymph or gentamicin administration attenuated this increase (Figure 3C). Besides, immunohistochemistry (IHC) confirmed the inhibition of autophagy in mammary glands by *E. coli* (Figure 3D). *E. coli* infection significantly decreased LC3 puncta while *Z. morio* hemolymph or gentamicin administration increased LC3 puncta (Figure 3D). These results suggest that *E. coli* inhibits ATG5/ATG16L1-mediated autophagy, and *Z. morio* hemolymph alleviates this inhibition.

### 2.4. Z. morio Hemolymph Repairs the Blood–Milk Barrier Integrity in E. coli-Induced Mastitis

**Figure 4:**
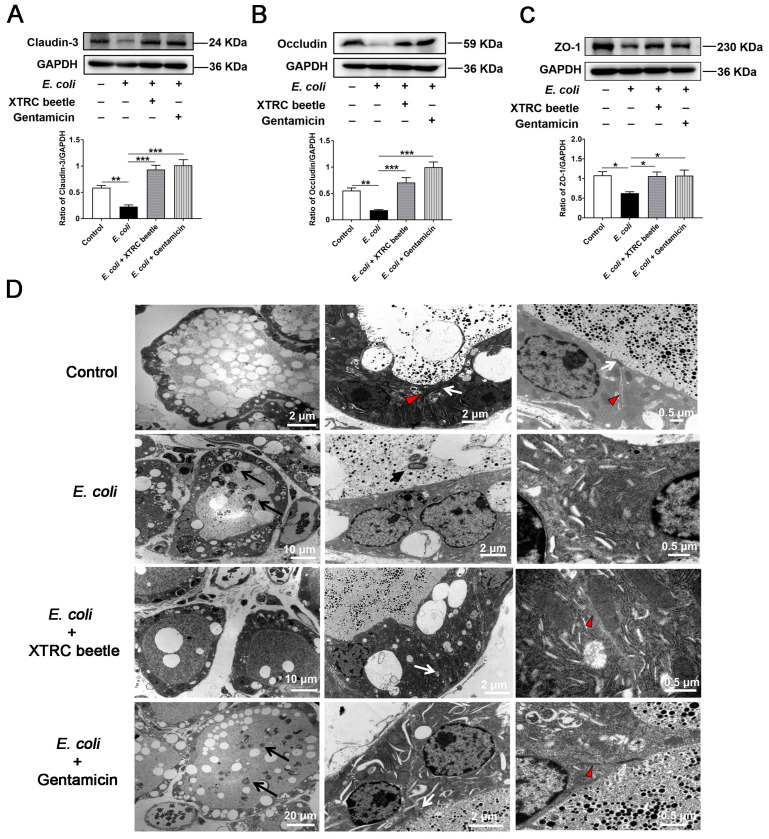
Effect of *Z. morio* hemolymph on the integrity of blood–milk barrier in *E. coli*-induced mastitis. (**A**–**C**) Western blotting analysis of Claudin3, Occludin and ZO-1 in mammary tissues. The panel shows the protein quantification using ImageJ software (version 1.50). (**D**) Representative transmission electron micrograph (TEM) images in each group. Microvilli are formed at the surface of luminal cells. MECs are connected by cell junctions. Long black arrows, neutrophils infiltration in acinar cavity; Short black arrows, *E. coli*; white arrows, desmosomes; red triangles, tight junctions (TJs). XTRC beetle stands for *Z. morio* hemolymph-treatment. Data presented are means ± SEM (*n* = 8 per group). * *p* < 0.05, ** *p* < 0.01, *** *p* < 0.001.

TJs are the basic structure of blood–milk barrier, which could restrict the invasion of microorganisms and regulate the exchange of various substances in the mammary gland. To elucidate the mechanism of *Z. morio* hemolymph on repairing the blood–milk barrier integrity, TJs were analyzed via western blotting and the transmission electron microscope (TEM). In the *E. coli* group, the protein level of Claudin-3 (Figure 4A), Occludin (Figure 4B) and ZO-1 (Figure 4C) was significantly lower than control group, while *Z. morio* hemolymph or gentamicin treatment group had an intense increase compared with the *E. coli* group (Figure 4A–C), indicating that *Z. morio* hemolymph repairs the TJs destroyed by *E. coli*. Consistent with the observation of H&E staining, TEM revealed *Z. morio* hemolymph significantly lower the thickening of the acinar walls and neutrophils infiltrated in the acinar cavity of the mammary gland (Figure 4D). As shown, TEM results also showed that the TJs were strengthened after *Z. morio* hemolymph or gentamicin treatment compared with the *E. coli* group, suggesting that *Z. morio* hemolymph repairs the tight junction destroyed by *E. coli*.

### 2.5. Z. morio Hemolymph Inhibits E. Coli-Induced Inflammatory Response in PMECs

**Figure 5:**
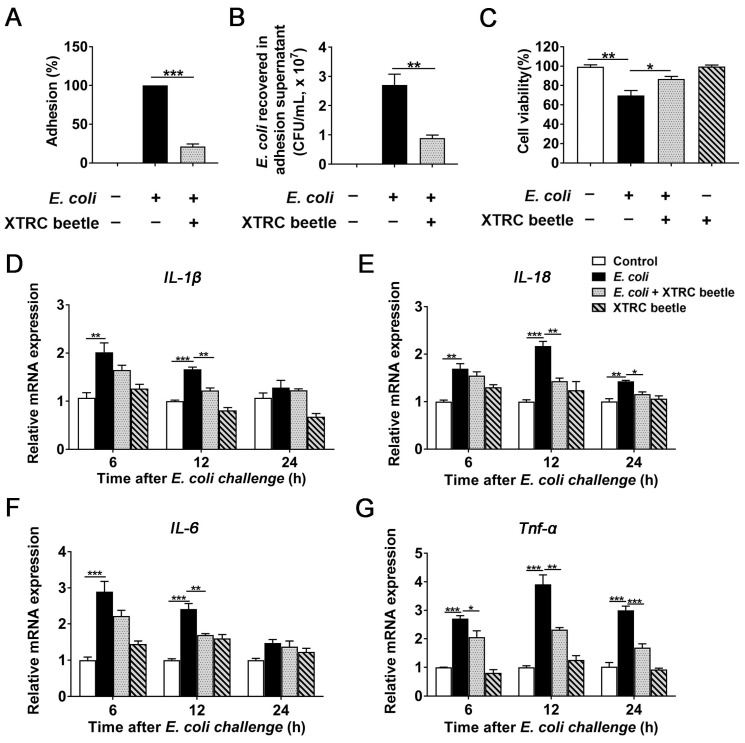
Effects of *Z. morio* hemolymph on *E. coli*-induced inflammatory response in PMECs. (**A**) The percentage of *E. coli* adhesions detected by adhesion assay of bacteria with PMECs. (**B**) CFU detection of *E. coli* in supernatants. (**C**) CCK-8 assay analysis of cell death of PMECs in each group at 12 h after *E. coli* infection. (**D**–**G**) The mRNA expression of *IL-1β, IL-18, IL-6* and *Tnf-α* in PMECs at 6, 12, 24 h post-infection. XTRC beetle stands for *Z. morio* hemolymph-treatment. Data presented are means ± SEM (*n* = 3 per group). * *p* < 0.05, ** *p* < 0.01, *** *p* < 0.001.

The ability of adhering to the cell surface is a prerequisite for bacterial infection. We explored the concentration of *E. coli* and *Z. morio* hemolymph in PMEC modle (Figure S2). To understand the influence of *Z. morio* hemolymph on the adhering activity of *E. coli*, plate counting assay was used to analyze the *E. coli* adhesion. At 12 h post-infection, the number of adherent *E. coli* was 1.76 × 10^7^ ± 3.14 × 10^6^ CFU (means ± SEM). Specifically, *Z. morio* hemolymph significantly reduced the percentage of adhering *E. coli* cells to 21.6% (Figure 5A). Moreover, the number of *E. coli* recovered in adhesion supernatant was determined, and the difference between hemolymph treatment and untreated groups was also obvious (Figure 5B), and the internalization of *E. coli* by PMECs was not observed. *Z. morio* hemolymph notably reduced the adhering activity of *E. coli*.

Cell viability of each group was analyzed by Cell Counting Kit-8 (CCK-8) assay to evaluate the effect of *Z. morio* hemolymph on cell death of PMEC induced by *E. coli*. Obviously, *E. coli* infection significantly reduced cell viability, while *Z. morio* hemolymph remarkably increased the viability of PMECs (Figure 5C).

Cytokines drive and regulate the development of inflammation. In our in vivo experiments, we have proven that *Z. morio* hemolymph have an anti-inflammatory role in *E. coli*-induced mice mastitis. To further this function of *Z. morio* hemolymph, with a particular focus on the expression of pro-inflammatory cytokines, the effect of *Z. morio* hemolymph on *E. coli*-induced inflammatory response in PMECs was examined by qRT-PCR assay. As shown in Figure 5, *E. coli* significantly increased mRNA expression of *IL-1β*, *IL-18*, *IL-6* and *Tnf-α* compared with control and *Z. morio* hemolymph (4 mg/mL) treatment groups, and *Z. morio* hemolymph markedly inhibited the increased mRNA expression of *IL-1β*, *IL-18*, *IL-6* and *Tnf-α* at 12 h after *E. coli* challenge (Figure 5D–G). These results further confirm the anti-inflammatory effect of *Z. morio* hemolymph.

### 2.6. Z. morio Hemolymph Suppresses E. Coli-Induced Activation of NLRP3 and Inhibition of ATG5/ATG16L1-Mediated Autophagy Signaling Pathway in PMECs

**Figure 6:**
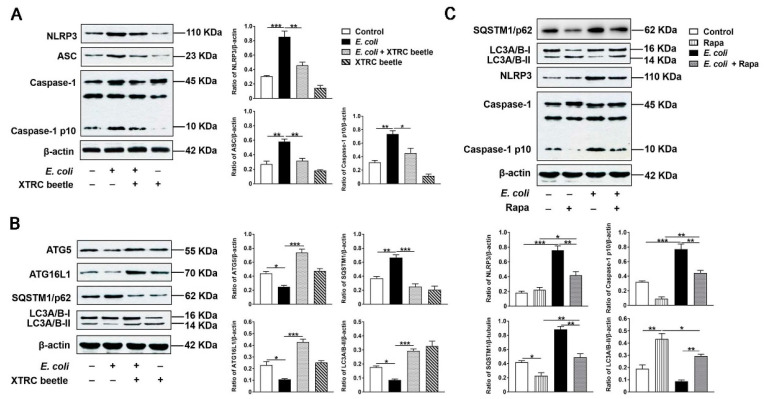
Effect of *Z. morio* hemolymph on the NLRP3 and ATG5/ATG16L1-mediated autophagy signaling pathway in *E. coli*-induced PMECs. (**A**) Western blotting analysis of NLRP3, ASC and Caspase-1. The right panel shows the protein quantification using ImageJ software (version 1.50). (**B**) Western blotting analysis of ATG5, ATG16L1, P62 and LC3A/B-II. The right panel shows the protein quantification using ImageJ software (version 1.50). (**C**) Western blotting analysis P62, LC3A/B-II, NLRP3 and Caspase-1 in PMECs after Rapa (1 μM) pretreatment. The right panel shows the protein quantification using ImageJ software (version 1.50). XTRC beetle stands for *Z. morio* hemolymph-treatment. Data presented are means ± SEM (*n* = 3 per group). * *p* < 0.05, ** *p* < 0.01, *** *p* < 0.001.

In vivo, we have found that *Z. morio* hemolymph inhibited the inflammatory response of mammary gland via down-regulating the NLRP3 signaling pathway activation and up-regulating autophagy activity. To further elucidate the anti-inflammatory mechanism of *Z. morio* hemolymph, NLRP3 and ATG5/ATG16L1-mediated autophagy signaling pathways were also detected in *E. coli*-induced PMECs. These results are consistent with in vivo. *E. coli* increased NLRP3, ASC and Caspase1 p10 expression levels, and inhibited ATG5, ATG16L1 and LC3A/B-II expression levels and increased P62 expression level, while *Z. morio* hemolymph altered such effects of *E. coli* (Figure 6A,B).

To clarify the effect of *E. coli* on autophagy in PMECs without considering other factors and try to simulate the protective mechanism of *Z. morio* hemolymph, the autophagy activator Rapa was introduced. As expected, in the presence of the Rapa, the expression of LC3A/B-II was up-regulated, and the expression of P62 was down-regulated (Figure 6C). Specially, Rapa displayed a potent inhibitory effect on NLRP3 and Caspase-1 activation in *E. coli*-induced PMECs (Figure 6C), indicating that activated autophagy suppresses NLRP3 inflammasome activation induced by *E. coli* to some extent. These results suggest that *Z. morio* hemolymph could inhibit inflammatory response by inhibiting NLRP3 signaling pathway and activating autophagy in *E. coli*-induced PMECs.

### 2.7. Z. morio Hemolymph Enhances the Protein Levels of Claudin3, Occludin and ZO-1 in PMECs

**Figure 7:**
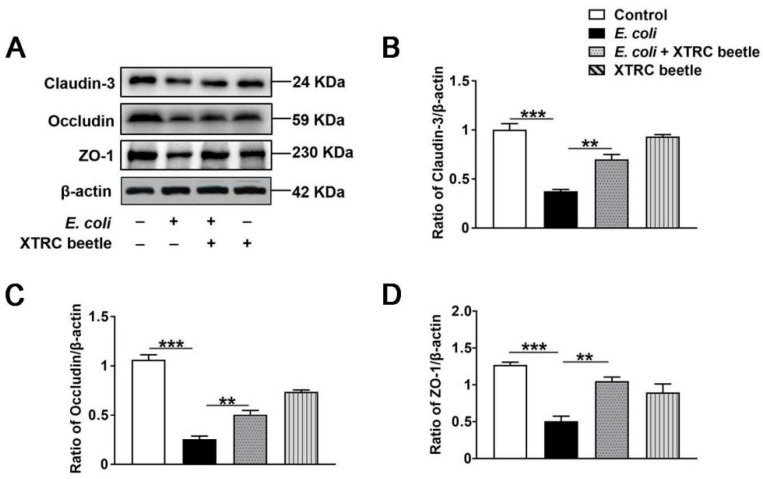
Effect of *Z. morio* hemolymph on the the protein levels of Claudin3, Occludin and ZO-1 in PMECs. (**A**) Expression of TJ proteins was determined by Western blotting. (**B**–**D**) Panel shows the protein quantification of TJ proteins using ImageJ software (version 1.50). XTRC beetle stands for *Z. morio* hemolymph-treatment. Data presented are means ± SEM (*n* = 3 per group). ** *p* < 0.01, *** *p* < 0.001.

In our in vivo experiments, we have found that *Z. morio* hemolymph repaired blood–milk barrier integrity by increasing the protein levels of Claudin3, Occludin and ZO-1. To further clarify whether *Z. morio* hemolymph increases the protein levels of TJs, PMECs were treated with 4 mg/mL *Z. morio* hemolymph for 12 h, and the protein levels of Claudin3, Occludin and ZO-1 were detected by Western blotting. As shown in Figure 7, *Z. morio* hemolymph remarkably increased the protein levels of Claudin3, Occludin and ZO-1 (Figure 7A–D).

## 3. Discussion

Coliform mastitis (CM) is a common clinical disease for sows. Currently, the traditional management of CM primary relies on antibiotics, which prone to drug residues and the emergence of new drug-resistant bacteria strains with significant side effects. Thus, developing a broad-spectrum, less residual anti-inflammatory drug for mastitis treatment is urgently needed. Our previous study has shown the antimicrobial impact of *Z. morio* hemolymph against mastitis pathogens [15]. In this study, we examined the effect and the mechanism of *Z. morio* hemolymph on mastitis in vivo and in vitro. Our findings demonstrated that *Z. morio* hemolymph could significantly alleviate mastitis via inhibiting inflammatory response and repairing the blood–milk barrier in *E. coli*-induced mastitis. The further mechanistic study found that *Z. morio* hemolymph significantly suppressed the NLRP3 signaling pathway activation and elevated autophagy activity in vivo and in vitro. These data suggested that *Z. morio* hemolymph might be a strong candidate for mastitis treatment.

The neutrophils and macrophages comprise the first line of defense against invading pathogens [29]. Once the invader is detected, mammary epithelial cells and macrophages will release chemoattractants that guide neutrophils to migrate to the area, and subsequent phagocytosis and killing of pathogens occur to exert a protective effect in the mammary gland [30]. CM is often accompanied by inflammatory cell infiltration in breast tissue [4]. According to the results of H&E staining, *E. coli* infection recruits large numbers of neutrophils into mammary alveolar spaces gland. Since bacterial toxins and oxidation products released from neutrophils can cause mammary tissue damage, rapidly eliminating of invading bacteria or reducing the number of effectively invading bacteria is necessary [30]. The adherence, colonization, and invasion of *E. coli* to mammary epithelial cells are prerequisites for intramammary infections [31]. In the present study, we detected the number of adherent *E. coli* in mammary gland and PMECs by plate counting assay in vivo and in vitro, respectively. Results obtained in this study reveal that *Z. morio* hemolymph can lessen the amount of effectively invading *E. coli* cells through reducing the adhering activity of *E. coli* and alleviate pathological injury of mammary gland in *E. coli*-induced mastitis.

As known, leukocytes constitute one of the vigorous defenses against exogenous infections in mammals [30]. When the body suffers from bacterial infection, the leukocytes would rapidly flow out of the blood and accumulate to the infected site to clear pathogenic bacteria [32]. In this study, we detected the change of peripheral blood parameters of mice and found that the number of leukocytes in peripheral blood was remarkably increased after *E. coli* challenge, while *Z. morio* hemolymphand decreased the number of leukocytes in peripheral blood. The immune balance of the body depends on the coordination and mutual restriction of T lymphocyte subsets. Thus, we also analyzed T lymphocyte subsets expression in peripheral blood. Remarkably, *Z. morio* hemolymph significantly reversed the reduction of CD3^+^CD4^+^ T cells induced by *E. coli,* but no substantial changes of CD3^+^CD8^+^ T cells was observed in each group, suggesting that *Z. morio* hemolymph was beneficial to the body to exert positive immune regulation. Additionally, *Z. morio* hemolymph significantly increased CD3^+^CD4^+^IL-2^+^ T levels and decreased CD3^+^TNF-α^+^ T levels, indicating that *Z. morio* hemolymph could ameliorate the inhibition effect of cellular immune function induced by *E. coli*. In this study, our results showed that *Z. morio* hemolymph could aid in the rapid recovery of inflamed mammary glands by modulating nonspecific and cellular immunity in mice.

LPS can elicit mastitis by *E. coli* in sows as well as in other mammal species [4,33] and trigger innate immune responses with activation of inflammasomes and release of proinflammatory cytokines [34]. The NLRP3 inflammasome has been known to play an important part role in many inflammatory diseases. However, dysregulated activation of NLRP3 inflammasome induces intense inflammation, leading to tissue damage [35,36]. In the current study, *E. coli* induces activation of NLRP3 inflammasomes (NLRP3, ASC and Casepase1), and release of IL-1β and IL-18. Additionally, *E. coli* also induced an up-regulation of pro-inflammatory cytokines (IL-6 and TNF-α), which are consistent with other studies on gene expression profiling in sows [1,4,37]. Studies have confirmed that proinflammatory cytokines attract neutrophils to recruit inflammatory areas via regulating adhesion molecules as well as chemokines in vascular endothelial cells [38]. Excessive neutrophils release of active substances, resulting in breast tissue damage. Interestingly, *Z. morio* hemolymph effectively inhibited the NLRP3 signaling pathway and the production of IL-6 and TNF-α. These results indicated that *Z. morio* hemolymph may regulate inflammatory responses through inhibiting the activation of NLRP3 inflammasome and production of proinflammatory cytokines.

Numerous lines of evidence have suggested that autophagy plays critical role in inflammation regulation [39]. ATG5/ATG16L1 signaling pathway has been reported to regulate autophagy [40]. In normal conditions, ATG5 and ATG16L1 are indispensable for the formation of the autophagosome, as well as for increasing autolysosome formation and autophagy flux. However, *E. coli* infection reduced ATG5 and ATG16L1 expression levels, further decreasing activity of autophagy, which mainly manifested as increased P62 expression level and decreased LC3A/B-II expression level. Interestingly, *Z. morio* hemolymph alleviated this inhibitory effect of *E. coli* on autophagy through regulating ATG5/ATG16L1 signaling pathway.

Recently, some studies have shown the cross-talk between autophagy and inflammasome activation [39,40,41]. Deletion of autophagy genes in mice has resulted in inflammasome-mediated IL-1β release and increased tissue damage [24,42]. Our previous study has demonstrated that decreased autophagy-related protein expression level and increased IL-1β and NLRP3 inflammasome-related protein expression level are involved in the pathogenesis of mastitis. To verify the regulatory effect of autophagy on NLRP3 inflammasomes during *E. coli* infection, Rapa, an autophagy activator, was introduced to the cell. As expected, Rapa elevated autophagy activity. Remarkably, Rapa displayed a potent inhibitory effect on the aberrant activation of NLRP3 inflammasome induced by *E. coli*. These results suggest that the inhibitory effect of *E. coli* on autophagy induces the aberrant activation of NLRP3 inflammasome, causing inflammatory injury. In contrast, autophagy inducer ameliorates inflammation of mammary gland. Consistent with this, *Z. morio* hemolymph also promoted autophagy activity and reduced *E. coli*-induced NLRP3 inflammasome activation in PMECs and in mice. *Z. morio* seems to be an autophagy inducer, exerting an anti-inflammatory effect by inhibiting NLRP3 inflammasome activation through activating autophagy. Collectively, our data indicate that *Z. morio* hemolymph limits detrimental inflammatory responses partly by regulating the NLRP3 inflammasome pathway through ATG5/ATG16L1-mediated autophagy pathway during *E. coli* infection.

The blood–milk barrier is a pivotal barrier for mammary gland to fight exogenous infections [43]. Tight junctions (TJs) constitute a vital structure of the blood–milk barrier, mainly preventing uncontrolled exchange between blood and milk [44]. In mastitis, LPS is reported to disrupt the integrity of the blood–milk barrier, which is usually associated with a breakdown of tight junction structure, further aggravating the condition [38]. In this study, we detected expression of the landmark proteins of TJs. We found that *E. coli* decreased Claudin3, Occludin and ZO-1 protein levels in vivo and in vitro, whereas *Z. morio* hemolymph increased such TJs protein levels. These data indicated that *Z. morio* hemolymph improved the integrity of blood–milk barrier through increasing the protein levels of TJs, suggesting that *Z. morio* hemolymph alleviated *E. coli*-induced mastitis at least partially by enhancing the blood–milk barrier. It confirmed that *Z. morio* hemolymph has a good effect on protecting the blood–milk barrier.

**Figure 8:**
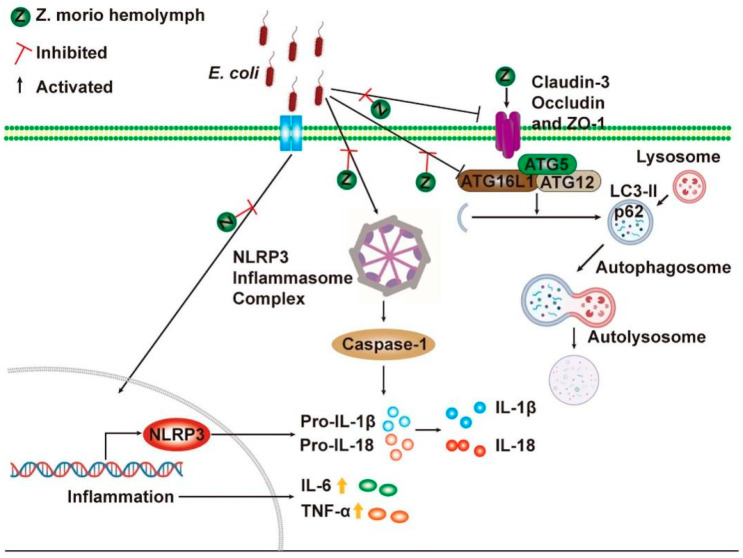
The mechanism of *Z. morio* hemolymph in anti-mastitis and improved blood milk barrier integrity. *Z. morio* hemolymph alleviates *E. coli*-induced inflammatory response of mammary gland through restraining NLRP3 signaling pathway and promoting ATG5/ATG16L1-mediated autophagy signaling pathway, and *Z. morio* hemolymph also enhances the integrity of blood-milk barrier via regulating the expression of TJs including Claudin-3, Occludin and ZO-1.

In summary, our study demonstrates that *Z. morio* hemolymph can alleviate inflammatory response through inhibiting NLRP3 inflammasome activation and enhancing autophagy activity, as well as repairing the blood–milk barrier to relieve *E. coli*–induced mastitis (Figure 8). All these results suggest that *Z. morio* hemolymph is a potential drug for mastitis treatment. These findings deepen understanding of insect antimicrobial peptides immune protection and contribute to its application in coliform mastitis prevention and treatment. The effective antibacterial mechanism and clinical application remain need to be further explored.

## 4. Materials and Methods

### 4.1. Animals

Pregnant Crl: CD1 (ICR) mice (10–12 weeks old, 30–35 g body weight) were purchased from the Beijing Vital River Laboratory Animal Technology Co., Ltd. (Beijing, China). The animals were housed in standard temperature conditions (24 ± 1 °C) with 12:12 h light-dark cycle and had ad libitum access to food and water. Randomization was used to assign samples to the experimental groups and to collect and process data.

### 4.2. Ethics Statement

All animal experiments in the study were performed in strict accordance with the Guidelines for Laboratory Animal Use and Care from the Chinese Center for Disease Control and Prevention and the Rules for Medical Laboratory Animals from the Chinese Ministry of Health, under protocol CAU20170812-6, approved by the Animal Ethics Committee of China Agricultural University. The pathogen (*E. coli*) was used in strict accordance with the Regulations on Biological Safety Management of Pathogen Microbiology Laboratory (000014349/2004-00195) from the State Council of the People’s Republic of China to avoid pathogen infection and transmission.

### 4.3. Establishment of Mouse Mastitis Model

32 lactating mice (3 days after birth of offspring) were divided into the following four groups (*n* = 8 per group) by randomization: the control group, *E. coli* (1 × 10^5^ CFU, 25 μL) treatment group, *E. coli* + *Z. morio* hemolymph (35 mg/mL, 25 μL) treatment group, *E. coli* + gentamicin (64 μg/mL, 25 μL) treatment group. The mice were anesthetized with Zoletil (55 mg/kg, Virbac, France). After that, the fourth inguinal mice mammary glands were treated with *E. coli* by canal injection, whereas the control group was similarly injected with an equal volume of sterile saline. *Z. morio* hemolymph or gentamicin was injected into the fourth inguinal mammary gland of mice following *E. coli* incubation, while mice in the control group and *E. coli*-treated group were given an equal volume of sterile saline, and again 12 h after. At 27 h post-infection (24 h after *Z. morio* hemolymph or gentamicin treatment), mice were euthanized, and the mammary glands were collected. The bacterial burden (the amount of *E. coli* recovered) in the mammary gland was measured on LB agar.

### 4.4. Bacteria Strains and Growth Conditions

*E. coli* CVCC1450 (EPEC, O111:K58) was purchased from China Institute of Veterinary Drug Center (Beijing, China). *E. coli* CAU15104 (ETEC/STEC, O3:H45) was isolated from the intestinal contents of diarrhea-weaned pigs in our laboratory. Bacteria were grown in Luria-Bertani (LB) broth (Oxoid, Basingstoke, England) overnight with shaking at 200 g at 37 °C, until reaching the mid-log phase (OD600 of 0.5).

### 4.5. Z. morio Immunization and Hemolymph Collection

The *Z. morio* immunization and hemolymph collection were conducted as previously described [15]. Briefly, 3rd instar larvae of *Z. morio* were injected with 1 μL of heat-killed, overnight culture of *E. coli* CVCC1450 (1 × 10^7^ cells per injection). At 24 h after *E. coli* challenge, the insects were chilled in ice water for 60 s, and then hemolymph (approximately 30 μL) was harvest into a precooled plastic tube by sectioning the metathoracic leg and squeezing the abdomen cavity gently. Boiling for 10 min, followed by a centrifugation (20,000× *g*, 30 min) at 4 °C. The cell-free hemolymph was clarified through a 0.2 μm filter, and 10 mg of hemolymph was run in 10% SDS-PAGE for a quality test. Cell-free hemolymph after inspection was then centrifugated (5000× *g*, 15 min) using an Amicon Ultra-30 centrifugal filter (Millipore, MA, U.S.A), the supernatant extract was collected for subsequent experiments. The hemolymph could be lyophilized and stored at −20 °C for long-term storage.

### 4.6. Cell Culture and Treatments

PMECs (a kind gift from Prof. Guoyao Wu in China Agricultural University) were maintained in Dulbecco’s Modified Eagle Medium/Ham’s F-12 medium (DMEM/F12) supplemented with 10% heat-inactivated fetal bovine serum (Thermo Scientific, Waltham, MA, USA), 5 μg/mL of insulin, 5 ng/mL of epidermal growth factor, 1 μg/mL of hydrocortisone, 50 μg/mL of gentamycin and 1 × PSN (penicillin-G, streptomycin, and neomycin) antifungal/antibiotics at 37°C in a 5% CO_2_ incubator for 24 h [45]. PMECs (4 × 10^5^ cells/well) were seeded onto 6-well cell culture plates and divided into four groups: the control group, *E. coli* (4 × 10^6^ CFU) treatment group, *E. coli* + *Z. morio* hemolymph (4 × 10^6^ CFU, 4 mg/mL) treatment group, *Z. morio* hemolymph (4 mg/mL) treatment group. At 12 h after *E. coli* infection, PMECs were collected for further analysis.

### 4.7. Histologic Assessment

The mammary tissues were fixed in 4% paraformaldehyde for at least 24 h, then placed in different concentrations of alcohol and xylene in turn, fixed with paraffin. The paraffin embedded tissues were sliced into 4 mm thick slices. For assessing histopathology changes, mammary tissues were stained with hematoxylin-eosin (H&E) and observed under a light-microscope as described previously [46].

### 4.8. Determination of Bacterial Load in the Mammary Gland

The mammary tissue abrasive solution was diluted 10 times continuously, and 10 μL tissue laps were obtained from each dilution and applied to the selective growth plate Eosin-Methylene Blue (EMB) agar (Aobox, Beijing, China). Each concentration was repeated 4 times. The plate was cultured at 37 °C in an atmosphere of 5% CO_2_ for 24 h. The bacterial load of *E. coli* was calculated according to the bacterial count results, which was quantified by measuring the colony-forming unit (CFU).

### 4.9. Enzyme-Linked Immunosorbent Assay (ELISA)

The concentrations of interleukin (IL)-1β and IL-18 in mammary tissues were measured by mouse specific commercially available ELISA kits (CSB-E08054m and CSB-E04609m; CUSABIO, Wuhan, Hubei, China). The experimental procedures were based on the manufacturer’s instructions.

### 4.10. Transmission Electron Microscopy (TEM)

Mammary tissue samples were cut into fragments of about 1 mm^3^ and fixed in 3% glutaraldehyde (pH 7.4) for 48 h at room temperature. The fixed tissues were post-fixed in 1% osmium tetroxide, dehydrated using a graduated ethanol series (30, 50, 70, 80, 90 and 100%), embedded in Epon (Energy Beam Sciences, Agawam, MA, USA), sliced into ultrathin sections (50–60 nm) using a Leica EM UC6 ultramicrotome (Leica Microsystems, Wetzlar, Germany) and stained with 3% uranyl acetate and lead citrate. The ultrathin sections of mammary tissues were observed using an H7500 transmission electron microscope (Hitachi, Tokyo, Japan).

### 4.11. Flow Cytometry

At 24 h after *E. coli* injection, a 500-μL aliquot of peripheral blood from each mouse was collected using Venoject glass tubes containing EDTA (Terumo Europe NV, Leuven, Belgium). Single-cell suspensions of peripheral blood was prepared as previously described. Different proportions of peripheral blood lymphocytes were assessed using CD3e/CD4/CD8/TNF-α/IL-2 triple-color flow cytometry. The following monoclonal antibodies were used: CD3e monoclonal antibody (Clone 145-2c11, FITC-conjugated, 11-0031-82; Thermo Fisher Scientific, Waltham, MA, USA), Rat anti-mouse CD4 (Clone GK 1.5, APC-Cy7–conjugated, 561830; BD Biosciences, San Jose, CA, USA), CD8 monoclonal antibody (clone 53–6.7, PerCP-Cy5.5–conjugated, 559585; Thermo Fisher Scientific, Waltham, MA, USA), IL-2 monoclonal antibody (clone JES-5H4, APC-conjugated, 17-7021-82; Thermo Fisher Scientific, Waltham, MA, USA) and TNF-α monoclonal antibody (clone MP6-XT22, PE-Cyanine7-conjugated, 25-7321-82; Thermo Fisher Scientific, Waltham, MA, USA). The stained cells were analyzed on a FACScalibur™ flow cytometer (BD Biosciences, San Jose, CA, USA), and data analysis was performed using FlowJo 9.3 software (Tree Star, Ashland, OR, USA).

### 4.12. Immunohistochemistry

The mammary tissues were fixed in 4% paraformaldehyde, embedded in paraffin and sectioned at 4-μm. The sections were rehydrated, treated with citrate buffer (10 mM, pH6) to exposure antigen, and incubated with 3% H_2_O_2_ for 30 min to eliminate peroxidase. After washing with PBS, the sections were blocked with 5% bovine serum albumin and incubated with rabbit polyclonal anti-LC3A/B (1:100 dilution, 12741) (Cell Signaling Technology, Danvers, MA, USA) at 4 °C overnight. After washing with PBS, the sections were incubated with HRP-conjugated goat anti-rabbit IgG (Zhongshan Golden Bridge Biotechnology Co., Beijing, China) at room temperature for 1 h and then were visualized with DAB Detection Kit (Zhongshan Golden Bridge Biotechnology Co., Beijing, China). Negative controls were performed using the same procedure with the exception of replacing the primary antibody with PBS and irrelevant rabbit serum in each batch. Images were captured using an Olympus BX41 microscope (Olympus, Tokyo, Japan).

### 4.13. Adhesion Assay

The adhesion assay was conducted as previously described [15]. Briefly, PMECs (4 × 10^5^ cells/well) were seeded into a 6-hole cell culture plate. Confluent cell monolayers were treated with *Z. morio* hemolymph (4 mg/mL) and *E. coli* CVCC1450 (4 × 10^6^ CFU). At 12 h after *E. coli* challenge, the monolayer cells were washed four times with PBS to remove non-adherent bacteria and then were harvested by 0.05% trypsin treatment for 10 min at 37 °C. The amount of *E. coli* recovered was cultured on LB agar and quantified by measuring colony-forming unit (CFU), as described above. An adhesion assay using *E. coli* alone served as a positive control (100% adhesion). The adhesion rate was defined as the adhered *E. coli* population on the PMECs treated with different conditions relative to the adhered *E. coli* population in the positive controls. The experiment was performed three independent times.

### 4.14. Internalization Assay

For the internalization assay, as previously described [15], PMECs were treated with *Z. morio* hemolymph (4 mg/mL), *E. coli* (4 × 10^6^ CFU) or *E. coli* + *Z. morio* hemolymph (4 × 10^6^ CFU + 4 mg/mL). At 12 h after treatment, the number of internalized *E. coli* was determined by adding 100 μg/mL of gentamicin to kill extracellular bacteria. The amount of *E. coli* recovered was cultured on the LB agar, and quantified by measuring colony-forming unit (CFU), as described above. The experiment was performed three independent times.

### 4.15. Cell Viability

The effect of *Z. morio* hemolymph on cell viability was determined using CCK8 assay. PMECs were treated with *Z. morio* hemolymph (4 mg/mL), *E. coli* (4 × 10^6^ CFU) or *E. coli* + *Z. morio* hemolymph (4 × 10^6^ CFU + 4 mg/mL) as described above. After that, 10 μL CCK-8 (Saint-Bio, Shanghai, China) was added to each well. After 2 h, absorbance (OD) was measured at 450 nm using a microplate reader.

### 4.16. Real-Time Quantitative PCR

Total RNA was extracted from PMECs for gene expression analysis using TRIzol reagent (Invitrogen, Carlsbad, CA, USA). An ABI 7500 real-time PCR system (Applied Biosystems, Foster City, CA, USA) was used for quantitative real-time PCR analyses. The sequences of the primers used were listed in Table 1. Relative mRNA expression data were shown as fold-change according to the 2^−ΔΔCT^ method as previously described [15]. Data of gene expression were normalized to the glyceraldehyde-3-phosphate dehydrogenase (Gapdh) gene. The experiment was performed three independent times.

### 4.17. Western Blotting

Proteins from mammary tissue samples were extracted for Western blotting assay. The following primary antibodies included rabbit polyclonal anti-NLRP3 (1:2000 dilution, 19771-1-AP), rabbit polyclonal anti-ASC (1:500 dilution, 10500-1-AP), rabbit polyclonal anti-ATG5 (1:1000 dilution, 10181-1-AP), rabbit polyclonal anti-ATG16L1 (1:1000 dilution, 19812-1-AP), rabbit polyclonal anti-sequestosome 1 (SQSTM1) (1:500 dilution, 18420-1-AP) (ProteinTech Group, Rosemont, IL, USA), rabbit polyclonal anti-Claudin-3 (1:500 dilution, abs130066) (Absin Bioscience, Shanghai, CHN), rabbit polyclonal anti-LC3A/B (1:1000 dilution, 12741) (Cell Signaling Technology, Danvers, MA, USA), rabbit polyclonal anti-Caspase-1 (1:1000 dilution, ab179515), rabbit polyclonal anti-ZO-1 (1:50 dilution, ab59720), and rabbit polyclonal anti-Occludin (1:8000 dilution, ab216327) (Abcam, Cambridge, UK). To verify equal sample loading, the membrane was incubated with mouse anti-β-actin (1:5000 dilution, 66009-1-Ig), mouse anti-GAPDH (1:5000 dilution, 60004-1-Ig) and rabbit anti-β-tubulin (1:1000 dilution, 10094-1-AP). HRP-conjugated anti-mouse IgG (1:5000 dilution, SA00001-1) or anti-rabbit IgG (1:5000 dilution, SA00001-2) (ProteinTech Group, Rosemont, IL, USA) were used as secondary antibodies.

### 4.18. Statistical Analysis

Using Prism 7 (GraphPad) to perform statistical analysis. Data were expressed as means ± SEM (*n* = 3 or 8). Student’s *t*-test and one-way analysis of variance (ANOVA) followed by the Tukey’s test were applied to analyze statistically significant differences at *p* < 0.05.

## Figures and Tables

**Table 1:** Real-time PCR primers.

Primers Name	Direction ^a^	Sequence (5′→3′)	Accession Number
IL-1β	F	GGCCGCCAAGATATAACTGA	NM_214055
	R	GGACCTCTGGGTATGGCTTTC	
IL-18	F	GCTGCTGAACCGGAAGACAA	NM_213997.1
	R	AAACACGGCTTGATGTCCCT	
IL-6	F	GGGAAATGTCGAGGCTGTG	NM_214399
	R	AGGGGTGGTGGCTTTGTCT	
Tnf-α	F	GCCCACGTTGTAGCCAATGTCAAA	NM_214022
	R	GTTGTCTTTCAGCTTCACGCCGTT	
Gapdh	F	CCAGAACATCATCCCTGCTT	NM_001206359
	R	GTCCTCAGTGTAGCCCAGGA	

^a^ F = forward; R = reverse.

## Data Availability

Not applicable.

## Supplementary Materials

The following supporting information can be downloaded at: https://www.mdpi.com/article/10.3390/ijms232113279/s1.
