# Supplementary material for: Z. morio Hemolymph Relieves E. coli-Induced Mastitis by Inhibiting Inflammatory Response and Repairing the Blood–Milk Barrier
Source: Int J Mol Sci. 2022 Oct 31;23(21):13279. doi: 10.3390/ijms232113279 (PMC9657162; doi:10.3390/ijms232113279)
Supplement: Supplementary file 1 [file ijms-23-13279-s001.zip › ijms-1968687-supplementary.pdf]

Supplementary Materials

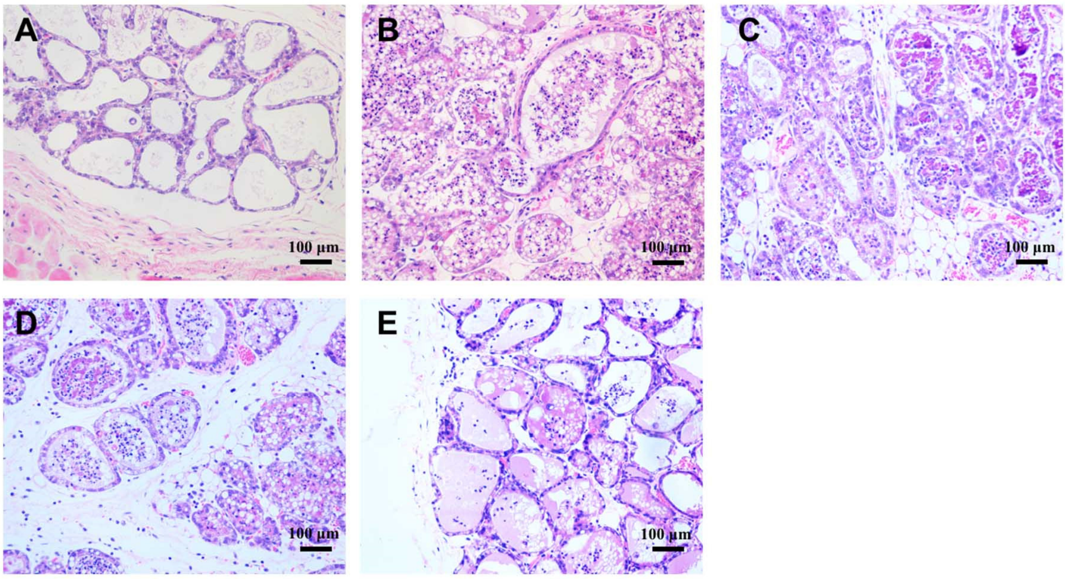

Figure S1. *Z. morio* hemolymph reduces pathological injury of mammary gland in *E. coli*-induced mastitis (original magnification 100 ×). (**A**) control group, (**B**) *E. coli* group, (**C**) *E. coli* + *Z. morio* hemolymph (15 mg/ml, 25 μL) group, (**D**) *E. coli* + *Z. morio* hemolymph (25 mg/ml, 25 μL) group, and (**E**) *E. coli* + *Z. morio* hemolymph (35 mg/ml, 25 μL) group.

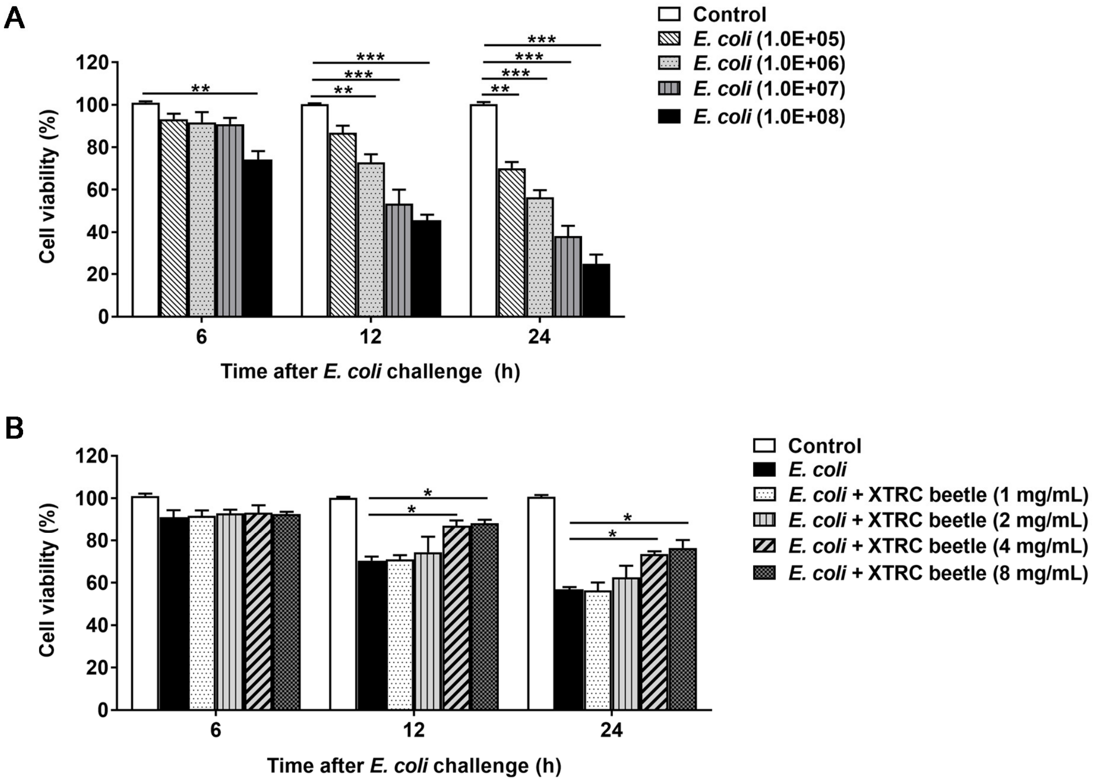

Figure S2. Determination of concentration of *E. coli* and *Z. morio* hemolymph in vitro. (**A**) PMECs were treated with different conditions of *E. coli* (1×10^5,1×10^6, 1×10^7, 1×10^8 CFU) for 6, 12, and 24 h, and cell viability was evaluated by CCK8 to determine the concentration of *E. coli*. (**B**) Cells were incubated with *E. coli* (1×10^6 CFU) and *Z. morio* hemolymph at 1, 2, 4, 8 mg/mL for 6, 12, and 24 h, respectively. And cell viability was evaluated by CCK8 to determine the working concentration of *Z. morio* hemolymph. * $p < 0.05$, ** $p < 0.01$, *** $p < 0.001$.
